# Modified Demirjian’s method for dental age estimation in Kosovar children and adolescents

**DOI:** 10.1007/s12024-025-01061-0

**Published:** 2025-08-16

**Authors:** Jeta Kelmendi, Rizky Merdietio Boedi, Marin Vodanovic, Donika Ilijazi Shahiqi, Bleron Azizi, Nikolaos Angelakopoulos

**Affiliations:** 1 Department of Orthodontics, Alma Mater Europaea, Campus Rezonanca, Pristina, Kosovo; 2 Department of Dentistry, Faculty of Medicine, Universitas Diponegoro, Semarang, Indonesia; 3 Department of Dental Anthropology, School of Dental Medicine, University of Zagreb, Zagreb, Croatia; 4 Department of Orthodontics AAB College, Pristina, Kosovo; 5 Department of Oral Surgery AAB College, Pristina, Kosovo; 6 Department of Orthodontics and Dentofacial Orthopedics, University of Bern, Bern, Switzerland

**Keywords:** Demirjian’s method, Dental age estimation, Normative values, Kosovar population

## Abstract

**Aim:**

The aim was to provide normative data on dental age estimation in the Kosovar population, to evaluate the relationship between customized maturity scores and the original Demirjian method, and to assess the accuracy of these scores in a sample of Kosovar children and adolescents.

**Materials and Methods:**

The study population consisted of 1106 digital panoramic radiographs randomly selected from 6- to 16-year-old patients treated at the University Dentistry Clinical Center of Kosovo. Only those images that were diagnostically acceptable, thus showing at least the left mandibular teeth, were included in the study to assess the developmental stage accurately.

**Results:**

Dental age estimates derived from the Kosovar normative tables were comparable to those based on maturity scores for the French-Canadian population. In girls aged 7.5–13 years and boys aged 7.5–12 years, dental maturity correlated strongly. However, the French-Canadian model overestimated age significantly compared to the Kosovar sample, where dental maturity started at about 6 years and peaked at 7 years. The Spearman’s rho of the relationship between dental ages determined by Demirjian’s method and maturity scores obtained from both populations was 0.997 for girls and 0.988 for boys. In conclusion, the present study demonstrates the need for population-specific adaptations of the Demirjian method and proves that modification of the method provides more reliable results when compared to the Kosovar population. The results indicate the possibility of further refinement of the Demirjian method for specific populations in order to improve the applicability and precision of the most commonly used method for age estimation.

**Supplementary Information:**

The online version contains supplementary material available at 10.1007/s12024-025-01061-0.

## Introduction

Research in dental age estimation (DAE) primarily seeks to have a more precise and efficient way to estimate chronological age, and dental development has long served as a variable to achieve this performance [1]. Accurate estimation of age holds critical importance across various disciplines, given its role as a key biological, psychological, and social indicator of transitional stages throughout the human lifespan [1, 2].

DAE plays a crucial role in multiple dental specializations. In orthodontics, early identification of late dental development can enhance treatment outcomes, especially in malocclusions associated with underlying skeletal discrepancies. Moreover, DAE holds significant relevance in pediatrics to assess dental growth. Additionally, in forensic odontology, it serves as a foundational tool for population analysis and contributes critically to the process of human identification [2–5]. As such, various methods have been developed to assess dental maturation in correlation with chronological age (CA).

Maturation refers to the progressive process culminating in the completion of physical development, with its stages assessable and illustrative through various straightforward methods [6]. Nonetheless, a universally accepted standard for the most accurate estimation of chronological age remains elusive. The most widely used method for age estimation today is based on examining the stages of tooth development and mineralization [7].

The most widely adopted method for assessing dental age in children was introduced by Demirijan in 1973 [7, 8]. This approach is based on the analysis of distinct stages of dental development, originally derived from a reference sample of French-Canadian children of Caucasian ethnicity. Demirjian’s method relies on the assessment of dental developmental stages, encompassing the progression from crown and root formation to apical closure [8]. The primary process of Demirjian method was to: (1) assign score to each seven primary mandibular teeth and then the sum of the score reflects the level of dental maturity and (2) the maturity score is subsequently converted into a dental age through the application of established reference tables.

It is generally known that the accuracy and generalizability of any age estimation methods must be evaluated across a broad spectrum of diverse populations [6, 8]. As such, notable discrepancies can emerge when applying these results to another population. Liversidge et al., have emphasized the need for developing population-specific dental age estimation models due to multiple variations that can appear in every population [9]. Such variations are likely attributable to the use of non-standardized statistical methodologies, manual adjustments of population reference curves, and inherent differences among populations in terms of lifestyle, environmental exposures, genetic background, sex, metabolism, and dietary habits [1].

Therefore, it is essential to assess the accuracy of the French-Canadian Caucasian standards used in Demirjian’s method, or alternatively, to adjust the reference tables for each specific population under analysis.

Assessing the precision and applicability of age estimation methods across diverse populations is essential. Therefore, the primary aim of this study was to establish normative data and maturation scores, or reference tables, specific to the Kosovar population. The secondary objective was to correlate previously obtained custom maturity scores with Demirjian’s original method [8] in a cohort of children and adolescents from Kosovo.

## Materials and methods

### Sample

A set of orthopantomograms (OPGs) from 1106 healthy male and female subjects, aged 6 to 16 years were used in this study (Table 1). The data were collected retrospectively from the database from the Radiology Unit at the University Dentistry Clinical Center of Kosovo (UDCCK) between March 2011 and August 2015, where OPGs were primarily taken for clinical care as part of the pre-treatment planning process. The research was submitted to the UDCCK’s Ethics and Research Committee for approval (Approval No. 292/15, November 2015) and was conducted following the ethical standards set by the Declaration of Helsinki (revised 2024) [10]. The subjects were registered anonymously, and their sex, date of birth, and date of X-ray acquisition were recorded for each OPG. None of the X-rays were taken specifically for the purposes of this research.

**Table 1:** Demographic distribution of the study sample

Age groups	Sex					
	Females		Males		Total	
6	15		12		27	
7	21		31		52	
8	47		49		96	
9	51		47		98	
10	63		49		112	
11	65		58		123	
12	61	(1)	48	(0)	109	(1)
13	66	(2)	66	(2)	132	(4)
14	70	(15)	64	(11)	134	(26)
15	52	(34)	54	(18)	106	(52)
16	52	(47)	65	(34)	117	(81)
**Total**	563	(99)	543	(65)	1106	(164)

Numbers in parentheses denote completed mineralization of all seven left mandibular teeth

### Inclusion and exclusion criteria

Radiographic images included in the study were required to be of diagnostic quality, clearly depicting all seven left mandibular teeth to allow for accurate assessment of their developmental stages. Radiographs that were blurred, exhibited geometric distortion, or had compromised tooth outlines due to technical errors were excluded. Additionally, radiographs were excluded if they showed bilaterally missing mandibular teeth or any signs of developmental anomalies. Only OPGs that fulfilled these inclusion criteria were selected for analysis. Inclusion criteria specifically required high-quality OPGs displaying all seven mandibular teeth in an intact and evaluable condition. The exclusion criteria comprised OPGs that exhibited tumors, the presence of surgical materials, mandibular or maxillary fractures, gross pathological findings, a history of orthodontic treatment, or evidence of infection in the mandibular region. OPGs of suboptimal image quality, which hindered accurate interpretation, were also excluded from the study. None of the X-rays were taken specifically for the purposes of this research. The children’s chronological age was calculated by subtracting the birth date from the date the OPG was taken, with the result rounded to two decimal places. Age groups were then categorized as one-year increments.

### Radiographic evaluation of mineralization

**Fig. 1:**
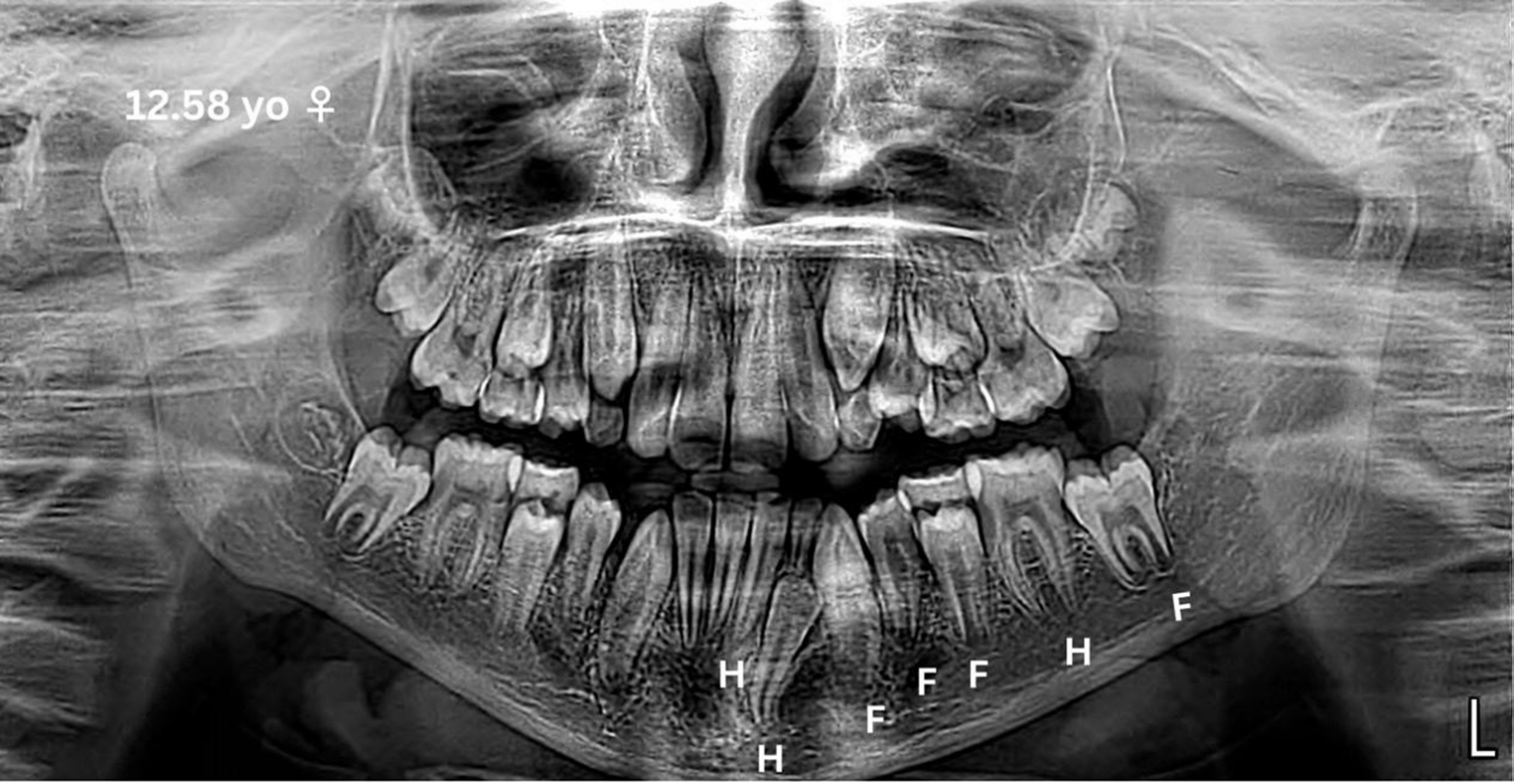
Example of mineralization staging with Demirjian’s method in a 12.58-year-old female subject

**Fig. 2:**
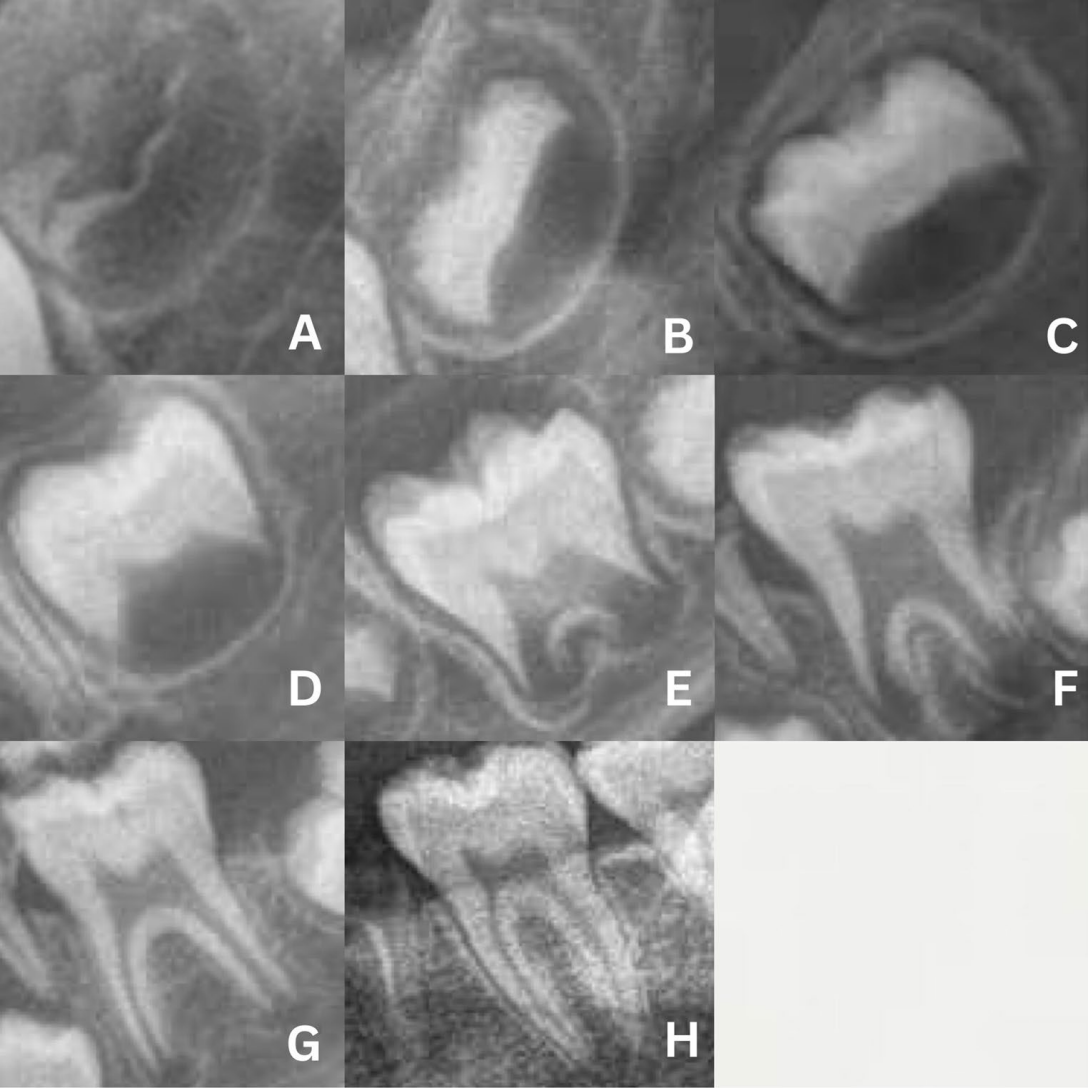
Mineralization stages of molars based on Demirjian’s method

Demirjian’s method involves three steps [8]. In the first step, the developmental stage of each tooth is identified. In the second step, maturity scores are assigned to each tooth. Finally, in the third step, these scores are converted into age (Figs. 1 and 2). The sum of the numerical values for all seven teeth is then compared with dental age tables, ranging from 0 to 100, separately for girls and boys. The study also provides graphical data that allows the maturity score to reflect the age interval at the 10th and 90th percentiles.

### Statistical analysis

Statistical analyses were computed using Microsoft Excel (MS 2010 Microsoft Corp., Redmond, WA, USA) and the Statistical Package for Social Science version 20.0 for Windows (SPSS Inc., Chicago, Illinois, USA) software. The time mineralization of stages of all evaluated teeth was presented as the mean, standard deviation, median, minimum, and maximum value. Inferential statistics consisted of CI, standard error, parametric and nonparametric tests. The statistical significance of the mean difference between chronological and estimated age was assessed using the paired-samples T-test and 95% confidence intervals (CI). The difference between the sexes for the same types of teeth was tested using the independent samples T-test. A one-sample T-test was used to compare chronological age with dental age, as estimated by Demirjian’s method, for both male and female Kosovar populations aged 6 to 16 years. Pearson’s correlation coefficient was used to evaluate the correlation between dental age, as determined by Demirjian’s method, and chronological age. Spearman’s correlation coefficient was used to assess the relationship between developmental stages according to Demirjian’s method and chronological age.

For each Demirjian stage (A–H) across the seven mandibular teeth (FDI 31 to 37), we calculated the average chronological age using a subset of 1106 healthy individuals (563 girls and 543 boys). These mean values were then assigned as maturity scores specific to the Kosovar population. The new maturity scores were calculated by using a median chronological age for each sex and stage. Furthermore, these scores were summed and incremented to the nearest increment of 0.1 years. To allow conversion to the dental age, these increments were treated as an ordinal variables, which were then modeled using a multi-class logistic regression function.

### Intra- and inter-rater reliability

Two trained evaluators independently assessed the developmental stages of the seven mandibular teeth; both were blinded to the subjects’ chronological age and sex. To assess intra-rater agreement, a random sample of 50 OPGs was re-evaluated by the first examiner after a three-month interval. For inter-rater agreement, the same set of OPGs was evaluated by the second examiner two weeks after the initial assessment. Cohen’s kappa values were calculated to assess agreement in mineralization stages within raters.

## Results

The maturity score for Kosovar children and adolescents was determined after estimating the dental age for the Kosovo population. The sample was evaluated using the three steps of Demirjian’s method, based on custom maturity scores. Maturity stages, ranging from A to H, were assigned to each tooth from FDI 31 to FDI 37, and the associated average chronological age was calculated based on a sample of 1,106 girls and boys from the Kosovar population (Table 1).

The obtained scores are presented in Table 2. For the Kosovar sample, the conversion of maturity scores into dental age was conducted according to Demirjian’s method, utilizing the average values of age groups interpolated with a logistic function. The results, along with the corresponding chronological ages ranging from 6 to 16 years in increments of 0.1 years, are shown in Table 3 for girls and Table 4 for boys. The abbreviations used are as follows: CI– central incisor, LI– lateral incisor, C– canine, PM1– first premolar, PM2– second premolar, M1– first molar, and M2– second molar.

**Table 2:** Demirjian’s dental maturity scores for girls and boys in a sample from Kosovo

Sex	Tooth	Stages							
		A	B	C	D	E	F	G	H
Girls	M2 (37)	4.9	7.4	8.3	9.5	10.6	12.0	13.3	15.3
	M1 (36)					5.9	5.8	8.5	12.6
	PM2 (35)	5.0	6.5	8.0	9.1	10.2	11.6	12.6	14.7
	PM1 (34)		4.8	7.4	8.3	9.7	11.0	12.1	14.3
	C (33)		5.5	6.1	7.9	8.7	10.1	12.1	14.1
	LI (32)				5.0	6.6	7.4	8.7	12.7
	CI (31)				5.2	6.6	6.7	7.7	12.2
Boys	M2 (37)		8.7	7.7	9.5	10.7	12.3	14.2	15.2
	M1 (36)					5.4	7.3	9.1	12.9
	PM2 (35)		8.5	7.4	8.2	9.7	11.6	13.5	14.8
	PM1 (34)			7.2	7.5	9.3	11.1	12.5	14.6
	C (33)			7.9	7.4	8.0	10.6	13.2	14.8
	LI (32)				8.4	7.1	8.2	9.2	13.1
	CI (31)					8.4	7.8	8.7	12.6

**Table 3:** Conversion of Demirjian’s maturity scores to dental age for girls in the Kosovo sample

Girls					
Age	Score	Age	Score	Age	Score
**6.0**	48.06	**10.0**	77.92	**14.0**	93.08
6.1	48.89	0.1	78.49	0.1	93.30
6.2	49.73	0.2	79.05	0.2	93.50
6.3	50.57	0.3	79.60	0.3	93.70
6.4	51.40	0.4	80.14	0.4	93.90
6.5	52.24	0.5	80.66	0.5	94.09
6.6	53.07	0.6	81.18	0.6	94.27
6.7	53.91	0.7	81.69	0.7	94.45
6.8	54.74	0.8	82.18	0.8	94.62
6.9	55.56	0.9	82.67	0.9	94.79
**7.0**	56.39	**11.0**	83.14	**15.0**	94.95
7.1	57.21	0.1	83.60	0.1	95.11
7.2	58.03	0.2	84.06	0.2	95.26
7.3	58.84	0.3	84.50	0.3	95.41
7.4	59.65	0.4	84.93	0.4	95.56
7.5	60.45	0.5	85.36	0.5	95.70
7.6	61.25	0.6	85.77	0.6	95.83
7.7	62.04	0.7	86.17	0.7	95.96
7.8	62.82	0.8	86.57	0.8	96.09
7.9	63.60	0.9	86.95	0.9	96.21
**8.0**	64.37	**12.0**	87.33	**16.0**	96.33
8.1	65.14	0.1	87.69		
0.2	65.89	0.2	88.05		
0.3	66.64	0.3	88.40		
0.4	67.38	0.4	88.74		
0.5	68.11	0.5	89.07		
0.6	68.83	0.6	89.39		
0.7	69.55	0.7	89.70		
0.8	70.25	0.8	90.01		
0.9	70.95	0.9	90.30		
**9.0**	71.63	**13.0**	90.59		
0.1	72.31	0.1	90.87		
0.2	72.97	0.2	91.15		
0.3	73.63	0.3	91.41		
0.4	74.27	0.4	91.67		
0.5	74.91	0.5	91.93		
0.6	75.53	0.6	92.17		
0.7	76.14	0.7	92.41		
0.8	76.75	0.8	92.64		
0.9	77.34	0.9	92.86		

**Table 4:** Conversion of Demirjian’s maturity scores to dental age for boys in the Kosovo sample

Boys					
Age	Score	Age	Score	Age	Score
**6.0**	45.46	**10.0**	79.58	**14.0**	94.80
6.1	46.41	0.1	80.20	0.1	94.99
6.2	47.37	0.2	80.81	0.2	95.17
6.3	48.34	0.3	81.40	0.3	95.34
6.4	49.30	0.4	81.98	0.4	95.51
6.5	50.27	0.5	82.54	0.5	95.67
6.6	51.23	0.6	83.09	0.6	95.83
6.7	52.19	0.7	83.62	0.7	95.98
6.8	53.15	0.8	84.14	0.8	96.13
6.9	54.11	0.9	84.65	0.9	96.27
**7.0**	55.07	**11.0**	85.15	**15.0**	96.40
7.1	56.02	0.1	85.63	0.1	96.54
7.2	56.97	0.2	86.10	0.2	96.66
7.3	57.91	0.3	86.55	0.3	96.78
7.4	58.85	0.4	86.99	0.4	96.90
7.5	59.78	0.5	87.42	0.5	97.02
7.6	60.70	0.6	87.84	0.6	97.13
7.7	61.62	0.7	88.25	0.7	97.23
7.8	62.53	0.8	88.64	0.8	97.33
7.9	63.43	0.9	89.02	0.9	97.43
**8.0**	64.32	**12.0**	89.40	**16.0**	97.53
8.1	65.20	0.1	89.76		
0.2	66.07	0.2	90.10		
0.3	66.93	0.3	90.44		
0.4	67.77	0.4	90.77		
0.5	68.61	0.5	91.09		
0.6	69.43	0.6	91.40		
0.7	70.25	0.7	91.70		
0.8	71.05	0.8	91.98		
0.9	71.83	0.9	92.26		
**9.0**	72.61	**13.0**	92.54		
0.1	73.37	0.1	92.80		
0.2	74.11	0.2	93.05		
0.3	74.85	0.3	93.30		
0.4	75.57	0.4	93.53		
0.5	76.27	0.5	93.76		
0.6	76.96	0.6	93.98		
0.7	77.64	0.7	94.20		
0.8	78.30	0.8	94.41		
0.9	78.95	0.9	94.61		

The number of samples with completed mineralization of the seven mandibular teeth on the left side, as seen in the OPGs from Kosovo, is presented in Table 1. Kappa coefficients for each tooth, along with the average rates for all seven teeth, are shown in Table 5. According to Altman, coefficients between 0.600 and 0.800 indicate good repeatability, which is the case in this study.

**Table 5:** Intraobserver repeatability of developmental stage estimation using Demirjian’s method in 50 orthopantomograms

Methods	Tooth							Mean
	FDI 31	FDI 32	FDI 33	FDI 34	FDI 35	FDI 36	FDI 37	
Demirjian’s method	0,787	0,857	0,643	0,696	0,622	0,783	0,790	0,740
Inter observer (kappa)	0.82	0.78	0.82	0.88	0.80	0.72	0.82	0.81

Overall findings are summarized in Table 6. Kosovar-specific maturity scores produced significantly different dental age estimates compared to chronological age in boys (mean difference = − 0.22 years) and girls (− 0.10 years). However, when the original French-Canadian scores were used, no statistically significant difference was observed. In addition, a close comparison between the two scoring methods indicated that the French-Canadian model overestimated dental age in the Kosovar sample. These observations further support the need for population-specific criteria. Further information on age- and sex-specific comparisons is provided in Tables S1–S3.

**Table 6:** Summary of the mean differences between dental age (DA) and chronological age (CA) based on Kosovar and French-Canadian maturity scores

Population	Sex	Mean DA– CA (years)	SD	95% CI	*p*-value
Kosovar	Female	–0.10	1.08	–0.18 to − 0.01	0.038
	Male	–0.22	1.21	–0.32 to − 0.12	< 0.001
French-Canadian	Female	+ 0.03	1.18	–0.07 to + 0.12	0.593
	Male	+ 0.03	1.26	–0.08 to + 0.14	0.589
Differences DA(D)– DA(DK)	Female	+ 0.12	0.44	+ 0.09 to + 0.16	< 0.001
	Male	+ 0.25	0.42	+ 0.21 to + 0.28	< 0.001

**DA(D)**– Dental age calculated using Demirjian’s method with maturity scores from the French-Canadian population;

**DA(DK)**– Dental age calculated using Demirjian’s method with maturity scores from the Kosovo sample;

**SD**– Standard deviation;

**95% CI**– 95% confidence interval of the difference;

**p**– Probability value for the difference under the null hypothesis, obtained using a one-sample t-test

**Fig. 3:**
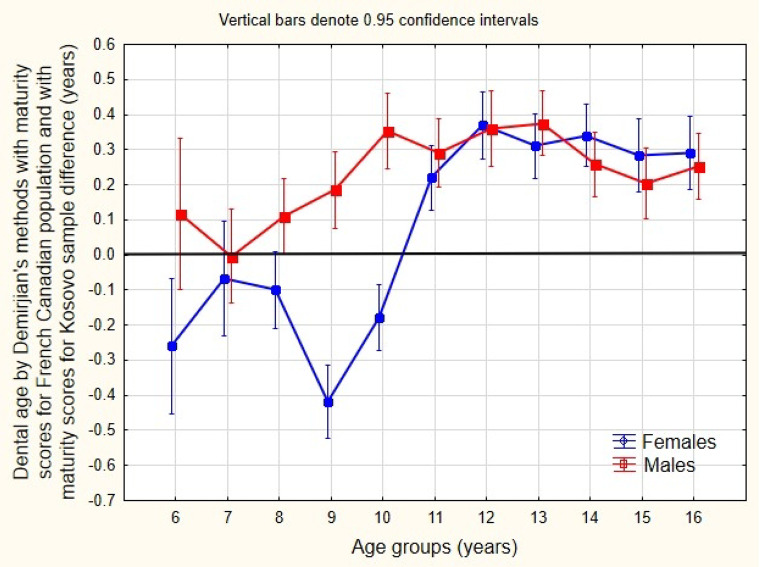
Box plot of the relationship between dental age by Demirjian’s method with maturity scores for a French Canadian population and with maturity scores for a Kosovo population, DA(D)– DA(DK). The box plot shows median and interquartile ranges, while the whiskers extend from box to highest and lowest values, extending outliners

Based on the results of the independent samples T-test, the difference in dental age between girls and boys, as estimated from their respective samples, was statistically significant for age groups 11 and 12 years (Table 6). The absolute deviation of dental age estimation from chronological age, as well as deviations for girls and boys, are shown in Table S2. The data reveal that the median deviation of dental age from chronological age for girls ranged from 0.45 to 1.14 years, while for boys, the range was from 0.44 to 1.35 years. These values are similar to or slightly lower than those observed in dental age estimations with maturity scores for the French-Canadian population. The frequency of absolute deviations between dental age and chronological age was assessed in half-year intervals. It is evident that, for both sexes, the frequency of deviations is most concentrated around the 0.5- to 1-year range, with the frequency gradually decreasing and becoming insignificant in the 3.5- and 4-year-old groups. As shown in the data in Table S2, statistically significant differences were found in both sexes among the 6-, 8-, and 16-year-old groups. Other significant differences were primarily found in boys, in the 7-, 10-, 11-, and 12-year-old groups, and in girls, within the 14-year-old group. These findings are illustrated in Fig. 3, where these deviations are evident as 95% confidence intervals (CIs) are completely or almost entirely below or above the line representing equal values of dental and chronological age (0.0 value).

**Fig. 4:**
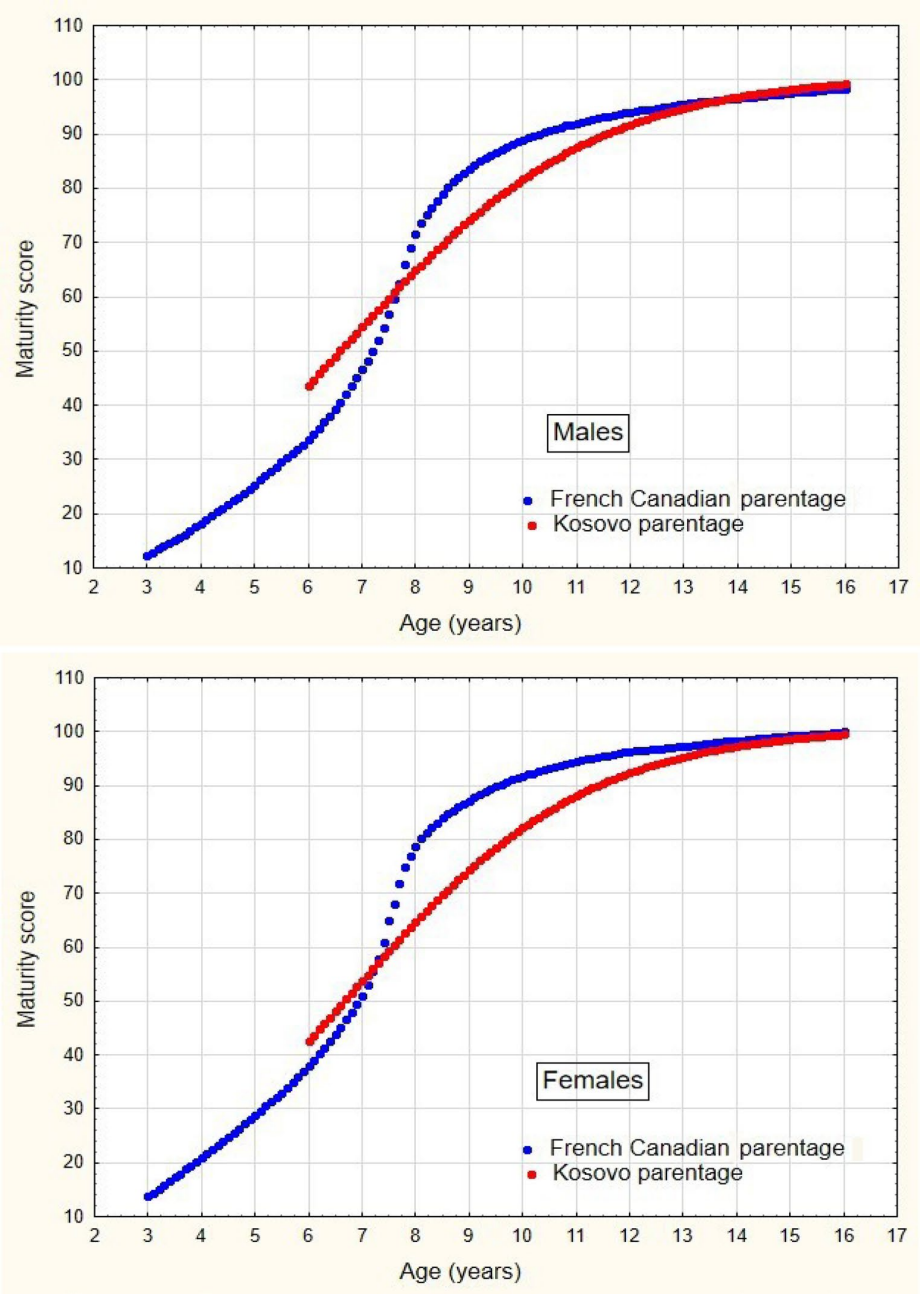
Median dental maturity scores by sex and population (French-Canadian and Kosovar) by chronological age

**Fig. 5:**
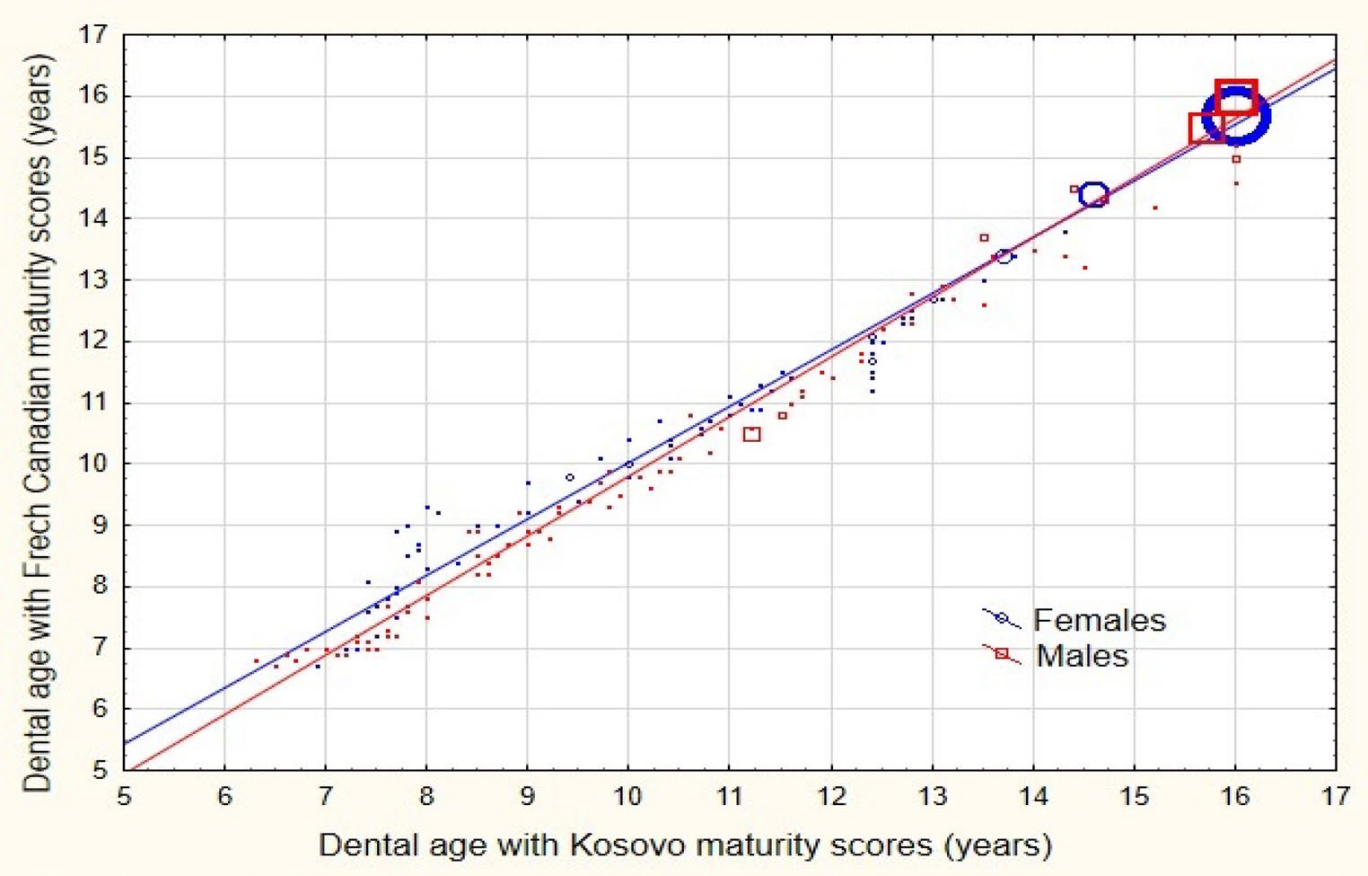
Scatterplot of dental age and maturity scores by Demirjian’s method for French-Canadian and Kosovar populations, by sex

As shown in Table S2, statistically significant differences were found in both sexes for the 6- and 16-year-old groups. Additional significant differences were found in girls in the 8-, 9-, 12-, and 15-year-old groups, and in boys in the 7- and 11-year-old groups. These data are illustrated in Fig. 4, where the differences are noticeable because 95% of the confidence intervals (CIs) are completely or almost entirely within the range below or above the line, which indicates equal values of dental and chronological age (0.0 value, highlighted by the thick line). A more precise depiction of the deviation from zero is presented in Fig. 5. Regarding the deviation from the 0.0 value, girls and boys exhibit similar trends, except for the 6-, 8-, 9-, and 10-year-old groups. Specifically, dental age with maturity scores for the Kosovo sample was overestimated in girls, while it was underestimated in boys when compared to the dental age estimation with maturity scores for the French Canadian population. From the 11th year onward, the dental age, along with its maturity scores, was estimated similarly for both sexes in the French Canadian population.

The absolute deviation of dental age, as evaluated using Demirjian’s maturity score method for the French Canadian population, is listed in Table S2. This deviation was assessed regardless of sex. It is evident that the median value, which divides the sample in half, for the deviation from chronological age ranged from 0.29 to 0.51 years for girls, while for boys, it ranged from 0.25 to 0.54 years.

As observed from the data in Table S3, a statistically significant deviation of 0.0 was found in both sexes for almost all age groups, as indicated by the results of the one-sample T-test. Due to the absence of normal distribution, the differences in the T-test results were further checked and confirmed using the bootstrap method. Differences close to zero were found in the 6-, 7-, and 8-year-old groups. These findings are illustrated in Fig. 3, where the deviations are clearly noticeable, as 95% of the confidence intervals (CIs) are completely or almost entirely within the range below or above the line denoting equal values of dental and chronological age. Individual deviations ranged from − 1.40 to 1.20 years for girls and from − 2.10 to 2.20 years for boys. The relationship between the maturity scores for the French Canadian population and the Kosovo sample is illustrated in Fig. 4. It is evident that the dental maturity of the French Canadian population in girls between 7.5 and 13 years of age and in boys between 7.5 and 12 years of age was significantly higher than the dental maturity observed in the Kosovo sample, which began at approximately age 6 and peaked at approximately age 7, and vice versa.

The correlation between dental age estimated by Demirjian’s method with maturity scores for the French Canadian population and maturity scores for the Kosovo population, as estimated by the nonparametric method (Spearman’s rho), was 0.997 (*n* = 563, *p* < 0.001) for girls and 0.988 (*n* = 543, *p* < 0.001) for boys. This strong correlation is clearly illustrated in the scatterplot in Fig. 5.

## Discussion

The results showed that the dental maturity of the Kosovar sample of both sexes, segmented with the modified Demirjian method, is comparable to that of other populations, with the same developmental alignments [3, 4]. Interestingly, the dental age estimation yielded comparable results with those obtained for the French-Canadian population [8]. However, discrepancies were observed when comparing the Kosovar sample to the French-Canadian group, particularly in the earlier stages of tooth development. These differences are likely attributable to a combination of genetic, environmental, and lifestyle factors that influence dental maturation across diverse populations [1]. Liversidge and colleagues, have highlighted that dental development varies significantly across populations, reinforcing the importance of creating localized standards [9]. Our findings support this, as applying French-Canadian standards resulted in consistent overestimations of dental age in younger Kosovar children. This discrepancy underlines the limitations of applying a single reference model to diverse populations.

The staging of tooth development aims to categorize the changes that occur along the continuous path of tooth growth, which serves as an indicator for age estimation. The most commonly used methods for age estimation are based on tooth development, which is divided into growing stages using dental radiographs [11]. In the present study, panoramic radiographs for children younger than 6 years were not included due to ethical and practical considerations, as radiologic exposure for this age group is difficult and rarely scientifically justified [12].

In particular, the Kosovar sample showed that dental maturity begins around age 6 and peaks around age 7, whereas the French-Canadian model tends to significantly overestimate age, especially in younger children. This highlights the importance of adapting reference tables to local populations to ensure more accurate age estimations.

The correlation between the dental age estimates from the Demirjian method and the maturity scores of both the Kosovar and French-Canadian populations was very high, with Spearman’s rho values of 0.997 for girls and 0.988 for boys. This strong correlation suggests that, while population-specific adaptations are necessary, the Demirjian method remains a robust tool for dental age estimation.

Many authors have reported different standards for dental maturation using various methods applied to diverse populations worldwide [13–15]. More recent studies have expanded this research to include additional populations across Europe and other regions [16–19].

Discrepancies between chronological age and estimated age, as reported in numerous studies using various methods, have prompted researchers to pursue more accurate approaches and develop new population-specific standards. With continuous advancements in computational modeling, the integration of such maturity scores into multicenter databases and artificial intelligence (AI) frameworks—particularly those based on convolutional neural networks—has emerged as a promising direction. AI-driven models have demonstrated near-perfect accuracy in automating dental age estimation from radiographs [19–21]. Normative datasets, such as the one provided in this study, could serve as valuable training inputs for these systems, potentially enhancing both the efficiency and precision of age estimation in clinical and forensic contexts.

As a result, new age prediction models and maturation scores for the Kosovar population were created based on the Demirjian method using multinomial functions, though these models require further validation. The current study found that Kosovo-specific maturation curves provide more accurate age estimations than the original Demirjian curves.

Consequently, in this study, the effectiveness of dental methods was compared in terms of the mean absolute difference between estimated and actual age, as well as the number of age estimates that were either < ± 1 year, which were considered accurate, or > ± 2 years, which were considered inaccurate [14]. It can be confirmed that Demirjian’s method was easy to apply and can be used in routine orthodontic evaluations and medical-legal cases to assess a person’s physiological age. While applying the French-Canadian standards, the Demirjian method slightly overestimates age in other populations. This finding aligns with results from different European and worldwide populations [19]. The most significant overestimation of these standards was observed in South Indian children [15]. Although Demirjian’s method has more population-specific studies available, the technique can be problematic, particularly in cases involving fragmented remains or when a tooth is bilaterally missing or malformed [18].

The Demirjian method has shown strong performance in terms of interobserver agreement and correlation between estimated dental age and chronological age. Its applicability has been widely tested across diverse populations of children aged 2 to 16.99 years, demonstrating consistent reliability and accuracy [22–29]. 

Ethnicity, environment, and secular changes could provide forensic anthropologists with erroneous results when calculating the estimated age for the biological profile in dental maturation in many populations [24, 25].

### Limitations and future directions of this research

The present study benefits from a substantial sample size, which enhances the reliability and representativeness of its findings within the Kosovar population. Although large sample sizes are traditionally recommended for studies of growth and development, previous literature has demonstrated that smaller samples may still yield valid outcomes when methodological rigor is maintained. Flood et al. noted that limited sample sizes could be appropriate for constructing dental maturity curves used in forensic age estimation [27]. In addition, Liversidge proposed that having at least ten individuals per age and sex group is adequate for generating accurate results while also minimizing the risk of age mimicry [28].

Nevertheless, the study presents several limitations. The decision to exclude third molars from the analysis may have restricted the assessment of dental maturity in older adolescents. Third molar development is typically considered a key indicator of late-stage dental maturation, and its exclusion may have reduced the study’s diagnostic comprehensiveness [3]. Furthermore, the analysis focused solely on the left mandibular teeth. While consistent with established practices in DAE, a more inclusive assessment involving both mandibular quadrants would likely provide a more representative overview of dental development.

The current study may benefit from a cross-validated test and training model design. Cross-validation (e.g., k-fold) or other resampling approaches should be conducted in future research to assess the stability and generalizability of the Kosovar-specific maturity scores both within and outside the current sample. Additionally, it is imperative that both general dental practitioners and forensic odontologists remain informed about the latest developments in DAE. Engaging with findings from large-scale population studies is particularly important, as such research typically yields more reliable statistical conclusions. Researchers should account for the effects of ancestral admixture, which may introduce variability in dental maturation patterns and, consequently, influence the accuracy of forensic age assessments [30].

Although the modified Demirjian method demonstrated effectiveness within the Kosovar population, further research is required to confirm its validity across different ethnic and regional groups in Kosovo. Comparative studies with normative data from neighboring populations would also be beneficial in identifying possible regional variations in dental development.

## Conclusions

The present study evaluated age estimation using the Demirjian method, with adaptations tailored to the Kosovar population. The findings underscore the critical need for population-specific reference tables to improve the precision of age estimation techniques. The enhanced accuracy observed in the Kosovar sample suggests that fine-tuning age estimation methods according to population-specific characteristics—shaped by genetic, environmental, and sociocultural factors—can lead to more reliable developmental assessments. These findings emphasize the necessity of population-specific calibrations for the Demirjian method, and other similar age assessment techniques, to improve both their accuracy and applicability. Such population-specific adjustments are crucial for advancing forensic age estimation, anthropological research, and clinical practice, providing more precise data for legal, medical, and archaeological purposes. The results suggest that future research should focus on developing population-specific age estimation models that can be broadly applicable across diverse ethnic and geographic groups.

### Key points

1. The modified method provides better age estimates for Kosovar children.
2. Notable discrepancies found between estimated and actual ages using the original method.
3. Findings support application in legal and clinical age assessments.

## Electronic supplementary material

Below is the link to the electronic supplementary material.

Supplementary Material 1

## Data Availability

The data supporting this study’s findings are available on request from the corresponding author.
