# Correction: Modified demirjian’s method for dental age estimation in Kosovar children and adolescents

**DOI:** 10.1007/s12024-025-01098-1

**Published:** 2025-10-20

**Authors:** Jeta Kelmendi, Rizky Merdietio Boedi, Marin Vodanovic, Donika Ilijazi Shahiqi, Bleron Azizi, Nikolaos Angelakopoulos

**Affiliations:** 1 Department of Orthodontics, Alma Mater Europaea, Campus Rezonanca, Pristina, Kosovo; 2 Department of Dentistry, Faculty of Medicine, Universitas Diponegoro, Semarang, Indonesia; 3 Department of Dental Anthropology, School of Dental Medicine, University of Zagreb, Zagreb, Croatia; 4 Department of Orthodontics AAB College, Pristina, Kosovo; 5 Department of Oral Surgery AAB College, Pristina, Kosovo; 6 Department of Orthodontics and Dentofacial Orthopedics, University of Bern, Bern, Switzerland

**Correction: Forensic Science, Medicine and Pathology** 10.1007/s12024-025-01061-0

In the original publication of this article, several errors were identified in the presentation of author details, affiliations, tables, figures, and captions. The following corrections have now been implemented:

 1. The envelope symbol indicating corresponding author status was incorrectly assigned to the first author. This has now been corrected.

2. The affiliation of the first author was previously listed as “Kosova” and has been corrected to “Pristina, Kosovo.” 

3. The affiliation of the second author was previously listed with the city “Zagreb,” which was incorrect. It has been corrected to “Semarang.”

4. Figures 1 and 2 were updated and replaced with revised versions to correct errors in the original publication.

5. The titles of Tables 1, 3, 4, and 5 were incorrect in the original publication and have now been corrected.

These corrections do not affect the study’s data, results, or conclusions. The original article has been corrected.

