# Supplementary material for: Modified Demirjian’s method for dental age estimation in Kosovar children and adolescents
Source: Forensic Sci Med Pathol. 2025 Aug 16;21(4):1743–54. doi: 10.1007/s12024-025-01061-0 (PMC12799705; doi:10.1007/s12024-025-01061-0)
Supplement: Supplementary file 1 — Supplementary Material 1 [file 12024_2025_1061_MOESM1_ESM.docx]

Table S1. Mean dental age and dental maturity scores estimated by Demirjian’s method in the Kosovar population, with chronological age and differences by age group and sex

| Age group | Sex | N | DA(DK) **–** CA | | | | | | |
| --- | --- | --- | --- | --- | --- | --- | --- | --- | --- |
|  |  |  | Mean | SD | 95% CI | | t statistics | | |
|  |  |  |  |  | Lower | Upper | t | Df | p |
| 6 | Female | 15 | 0.84 | 0.76 | 0.42 | 1.26 | 4.297 | 14 | 0.001 |
|  | Male | 12 | 1.05 | 0.48 | 0.74 | 1.35 | 7.624 | 11 | < 0.001 |
| 7 | Female | 21 | 0.23 | 1.04 | -0.25 | 0.70 | .996 | 20 | 0.331 |
|  | Male | 31 | 0.47 | 0.73 | 0.20 | 0.73 | 3.565 | 30 | 0.001 |
| 8 | Female | 47 | -0.40 | 0.81 | -0.64 | -0.16 | -3.383 | 46 | 0.001 |
|  | Male | 49 | -0.29 | 0.75 | -0.51 | -0.07 | -2.703 | 48 | 0.009 |
| 9 | Female | 51 | -0.10 | 0.94 | -0.36 | 0.16 | − .757 | 50 | 0.453 |
|  | Male | 47 | -0.10 | 0.83 | -0.34 | 0.15 | − .812 | 46 | 0.421 |
| 10 | Female | 63 | -0.16 | 1.07 | -0.43 | 0.11 | -1.213 | 62 | 0.230 |
|  | Male | 49 | -0.25 | 0.75 | -0.46 | -0.03 | -2.301 | 48 | 0.026 |
| 11 | Female | 65 | 0.00 | 1.07 | -0.26 | 0.27 | .034 | 64 | 0.973 |
|  | Male | 58 | -0.91 | 1.13 | -1.20 | -0.61 | -6.117 | 57 | < 0.001 |
| 12 | Female | 61 | 0.22 | 1.25 | -0.10 | 0.54 | 1.397 | 60 | 0.168 |
|  | Male | 48 | -0.74 | 1.40 | -1.15 | -0.33 | -3.655 | 47 | 0.001 |
| 13 | Female | 66 | -0.01 | 1.28 | -0.32 | 0.31 | − .059 | 65 | 0.953 |
|  | Male | 66 | -0.03 | 1.58 | -0.42 | 0.35 | − .178 | 65 | 0.859 |
| 14 | Female | 70 | -0.35 | 1.30 | -0.66 | -0.04 | -2.271 | 69 | 0.026 |
|  | Male | 64 | 0.05 | 1.62 | -0.36 | 0.45 | .242 | 63 | 0.810 |
| 15 | Female | 52 | 0.02 | 0.88 | -0.23 | 0.26 | .147 | 51 | 0.883 |
|  | Male | 54 | 0.05 | 1.19 | -0.27 | 0.38 | .328 | 53 | 0.744 |
| 16 | Female | 52 | -0.50 | 0.51 | -0.64 | -0.36 | -7.161 | 51 | < 0.001 |
|  | Male | 65 | -0.48 | 0.66 | -0.65 | -0.32 | -5.865 | 64 | < 0.001 |
| 6–16 | Female | 563 | -0.10 | 1.08 | -0.18 | -0.01 | -2.084 | 562 | 0.038 |
|  | Male | 543 | -0.22 | 1.21 | -0.32 | -0.12 | -4.251 | 542 | < 0.001 |

Table S2. Mean dental age and dental maturity scores estimated by Demirjian’s method in the French-Canadian population, with chronological age and differences by age group and sex

| Age group | Sex | N | DA(D) **–** CA | | | | | | |
| --- | --- | --- | --- | --- | --- | --- | --- | --- | --- |
|  |  |  | Mean | SD | 95% CI | | t statistics | | |
|  |  |  |  |  | Lower | Upper | T | df | p |
| 6 | Female | 15 | 0.58 | 0.96 | 0.05 | 1.11 | 2.333 | 14 | 0.035 |
|  | Male | 12 | 1.16 | 0.69 | 0.73 | 1.60 | 5.879 | 11 | < 0.001 |
| 7 | Female | 21 | 0.16 | 0.88 | -0.24 | 0.56 | 0.823 | 20 | 0.420 |
|  | Male | 31 | 0.46 | 0.60 | 0.24 | 0.68 | 4.275 | 30 | < 0.001 |
| 8 | Female | 47 | -0.50 | 0.63 | -0.68 | -0.31 | -5.391 | 46 | < 0.001 |
|  | Male | 49 | -0.18 | 0.76 | -0.40 | 0.04 | -1.664 | 48 | 0.103 |
| 9 | Female | 51 | -0.52 | 1.08 | -0.82 | -0.22 | -3.432 | 50 | 0.001 |
|  | Male | 47 | 0.09 | 0.91 | -0.18 | 0.35 | 0.649 | 46 | 0.520 |
| 10 | Female | 63 | -0.34 | 1.36 | -0.68 | 0.00 | -1.983 | 62 | 0.052 |
|  | Male | 49 | 0.11 | 0.92 | -0.16 | 0.37 | 0.810 | 48 | 0.422 |
| 11 | Female | 65 | 0.22 | 1.36 | -0.11 | 0.56 | 1.332 | 64 | 0.187 |
|  | Male | 58 | -0.62 | 1.42 | -0.99 | -0.24 | -3.307 | 57 | 0.002 |
| 12 | Female | 61 | 0.59 | 1.30 | 0.26 | 0.93 | 3.574 | 60 | 0.001 |
|  | Male | 48 | -0.38 | 1.62 | -0.85 | 0.09 | -1.618 | 47 | 0.112 |
| 13 | Female | 66 | 0.30 | 1.29 | -0.02 | 0.62 | 1.986 | 65 | 0.062 |
|  | Male | 66 | 0.34 | 1.65 | -0.06 | 0.75 | 1.679 | 65 | 0.098 |
| 14 | Female | 70 | -0.01 | 1.24 | -0.31 | 0.28 | -0.093 | 69 | 0.927 |
|  | Male | 64 | 0.31 | 1.51 | -0.07 | 0.68 | 1.626 | 63 | 0.109 |
| 15 | Female | 52 | 0.30 | 0.89 | 0.05 | 0.55 | 2.451 | 51 | 0.018 |
|  | Male | 54 | 0.26 | 1.19 | -0.07 | 0.58 | 1.591 | 53 | 0.117 |
| 16 | Female | 52 | -0.21 | 0.53 | -0.36 | -0.06 | -2.887 | 51 | 0.006 |
|  | Male | 65 | -0.23 | 0.61 | -0.38 | -0.08 | -3.042 | 64 | 0.003 |
| 6–16 | Female | 563 | 0.03 | 1.18 | -0.07 | 0.12 | 0.535 | 562 | 0.593 |
|  | Male | 543 | 0.03 | 1.26 | -0.08 | 0.14 | 0.540 | 542 | 0.589 |

DA(D) **–** CA – Difference between dental age (estimated using Demirjian’s method) and chronological age, based on maturity scores from the French-Canadian and Kosovar populations;

SD – Standard deviation;

95% CI – 95% confidence interval of the difference;

p – Probability value for the difference under the null hypothesis, obtained using a one-sample t-test.

Table S3. Mean dental age and dental maturity scores estimated by Demirjian’s method for the French-Canadian and Kosovar populations, with differences by age group and sex

| Age group | Sex | N | DA(D) **–** DA(DK) | | | | | | |
| --- | --- | --- | --- | --- | --- | --- | --- | --- | --- |
|  |  |  | Mean | SD | 95% CI | | t statistics | | |
|  |  |  |  |  | Lower | Upper | t | df | P |
| 6 | Female | 15 | -0.26 | 0.46 | -0.52 | 0.00 | -2.170 | 14 | 0.048 |
|  | Male | 12 | 0.12 | 0.30 | -0.07 | 0.31 | 1.356 | 11 | 0.202 |
| 7 | Female | 21 | -0.07 | 0.41 | -0.25 | 0.12 | − .747 | 20 | 0.464 |
|  | Male | 31 | 0.00 | 0.32 | -0.12 | 0.11 | − .056 | 30 | 0.956 |
| 8 | Female | 47 | -0.10 | 0.37 | -0.21 | 0.01 | -1.852 | 46 | 0.070 |
|  | Male | 49 | 0.11 | 0.29 | 0.03 | 0.19 | 2.657 | 48 | 0.011 |
| 9 | Female | 51 | -0.42 | 0.51 | -0.56 | -0.28 | -5.913 | 50 | < 0.001 |
|  | Male | 47 | 0.19 | 0.28 | 0.10 | 0.27 | 4.499 | 46 | < 0.001 |
| 10 | Female | 63 | -0.18 | 0.49 | -0.30 | -0.05 | -2.857 | 62 | 0.006 |
|  | Male | 49 | 0.35 | 0.29 | 0.27 | 0.44 | 8.454 | 48 | < 0.001 |
| 11 | Female | 65 | 0.22 | 0.49 | 0.10 | 0.34 | 3.627 | 64 | 0.001 |
|  | Male | 58 | 0.29 | 0.48 | 0.16 | 0.42 | 4.571 | 57 | < 0.001 |
| 12 | Female | 61 | 0.37 | 0.30 | 0.29 | 0.45 | 9.551 | 60 | < 0.001 |
|  | Male | 48 | 0.36 | 0.50 | 0.21 | 0.51 | 4.955 | 47 | < 0.001 |
| 13 | Female | 66 | 0.31 | 0.28 | 0.24 | 0.38 | 9.114 | 65 | < 0.001 |
|  | Male | 66 | 0.38 | 0.53 | 0.24 | 0.51 | 5.728 | 65 | < 0.001 |
| 14 | Female | 70 | 0.34 | 0.15 | 0.30 | 0.38 | 19.116 | 69 | < 0.001 |
|  | Male | 64 | 0.26 | 0.34 | 0.17 | 0.34 | 6.145 | 63 | < 0.001 |
| 15 | Female | 52 | 0.28 | 0.05 | 0.27 | 0.30 | 41.017 | 51 | < 0.001 |
|  | Male | 54 | 0.20 | 0.37 | 0.10 | 0.30 | 4.073 | 53 | < 0.001 |
| 16 | Female | 52 | 0.29 | 0.03 | 0.28 | 0.30 | 70.344 | 51 | < 0.001 |
|  | Male | 65 | 0.25 | 0.48 | 0.13 | 0.37 | 4.256 | 64 | < 0.001 |
| 6–16 | Female | 563 | 0.12 | 0.44 | 0.09 | 0.16 | 6.619 | 562 | < 0.001 |
|  | Male | 543 | 0.25 | 0.42 | 0.21 | 0.28 | 13.951 | 542 | < 0.001 |

DA(D) **–** DA(DK) – Difference in dental age estimated using Demirjian’s method with maturity scores from the French-Canadian population (DA(D)) and the Kosovar population (DA(DK));

SD – Standard deviation;

95% CI – 95% confidence interval of the difference;

p – Probability value for the difference under the null hypothesis, obtained using a one-sample t-test.
